# Phrases and Realities

**Published:** 1918-04-13

**Authors:** 


### PHRASES AND REALITIES.

The war is proving itself a testing-school for phrases. Words and formulas of heroic mould are in increasing measure feeling the stress of inquisition, and their value, or their worthlessness, is being tried as by fire. High professions and lofty sentiments have to face stern realities, and their significance must be judged by the fashion in which they meet the strain. Certain phrases which were very hard worked in the early days have long since vanished into nothingness, and to recall them now would be very like cruelty. The process has been a gradual one but it would seem that at long last we have come to a position in which the true meaning of the past four years cannot possibly be avoided. This meaning has been covered up by dignified phrases and diplomatic subtleties, and certain minds have clung to these as justifying the belief that the whole thing has been due to an unhappy misunderstanding, and that if only a few lofty persons would meet and talk together everything would be nicely and pleasantly arranged. Now, thanks to the frankness of certain disengaged diplomatists, even the blind must see that, not accident, but deliberate choice, determined the war, and that the plan thus conceived must suffer either a full realisation or a complete defeat. Recent events on the battlefield have emphasised this position, and the smooth and comfortable formulas which proposed solutions other than by fighting and enduring have ceased to find an audience, and the occasional orator who clings to them is a mere voice in the wilderness. The ambition to rule the world and the will to victory are the hard facts which are displayed to us, and the smooth professions which for long concealed them are now out of sight—they are phrases which have faded before realities. The education has been a slow one, but it may now be considered practically complete so far as the British public is concerned. The pleasing possibilities sketched by the artist in words are seen to owe their inspiration not to facts, but to fancies, and the admirers of these amiable enterprises recognise that they have been as ill-prepared for the future as they have been ill-informed about the past. They have had many pleasant afternoons; but these have not well-equipped them for the necessities of a working day.

The lesson, it must be allowed, is a severe one, and its severities are far from being exhausted. Yet the discipline may prove salutary provided it is applied over the full field which it commands. It is so easy to be satisfied with words and ready assurances, and the desire for certainty is strong. Hence the talker and the phrase-maker have always ready hearers, and in normal times their triumphs are hardly challenged. But sooner or later in life stress arises, and facts and realities assert themselves. The experience, however, may well be too late to be of value to the individual judgment, and it is here that a true scheme of education finds its value. To cultivate the faculty of distinguishing between seeming and being is an ambition for the individual as well as for the nation, and this in substance means an ability to recognise realities and a determination not to be satisfied with words. Such a path is not an easy one. But it is free from illusions, and it is prepared against surprises. Perhaps some of our educational authorities advise it and pursue it. But not a few cultivate quite a different method. The war has inflicted some severe blows on certain of the idols of the market-place, and if it gave all of us the courage and resolution to face realities and to eschew pretence there would be at least a measure of compensation for its horror and havoc.

